# Dr. C. H. Metgud

**Published:** 2010

**Figure:**
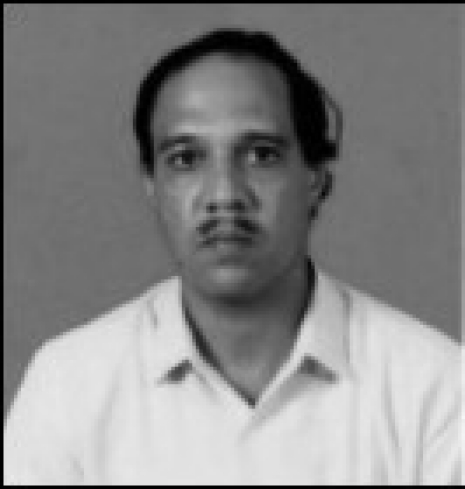
30^th^ June 1956 – 06^th^ May 2010

Dr. C. H. Metgud completed MBBS in the year 1980, DA in 1984, and MD Anaesthesiology in 1990, from J. N. medical College, Belgaum (Karnataka University, Dharwad). From 1991 to 2005, he worked as teaching faculty of Anaesthesiology holding various hierarchical positions at J. N. Medical College, Belgaum. From July 2005 to May 2010, he served as Professor and Head of Department of Anaesthesiology at Belgaum Institute of Medical Sciences, Belgaum, and played a pivotal role in setting up the Department of Anaesthesia and ancillary departments in newly established Belgaum Institute of Medical Sciences, Belgaum. To his credit, there are 21 publications at international/ national level. He had also delivered guest lectures at various platforms on various topics of anaesthesiology. He was an active member of ISA Belgaum city branch. May his soul rest in peace.

