# CORRIGENDUM

**Published:** 2010

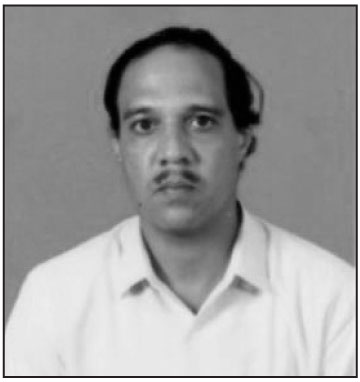



Indian Journal of Anaesthesia Mar-Apr 2010; Vol. 54; Issue 2

Article Type: Obituary

Page 156; Column: Bottom of the page; Title Heading and First line

Dr. Chinnappa S. Metgud

Should be read as

Dr. C H Metgud

The error is regretted.

Editor, Indian Journal of Anaesthesia

